# Factors Associated With Pre-hospital Delay in Patients With Acute Myocardial Infarction

**DOI:** 10.5812/ircmj.2367

**Published:** 2013-04-05

**Authors:** Hossein Farshidi, Shafei Rahimi, Ahmadnoor Abdi, Sarah Salehi, Abdoulhossain Madani

**Affiliations:** 1 Hormozgan Cardiovascular Research Center, Hormozgan University of Medical Sciences, IR Iran

**Keywords:** Myocardial Infarction, Pre-Hospital Delay, Education

## Abstract

**Background:**

Treatment of patients with acute myocardial infarction (AMI) is time related, so delay in treatment could affect prognosis. Recognizing pre-hospital or in-hospital delays in initiating treatment and reducing these factors is very efficacious in treatment of these patients.

**Objectives:**

The aim of this study is evaluate the causes of pre-hospital delay just as other studies on effect of different variables such as socioeconomic and personal factors on pre-hospital delay in with patients with AMI.

**Materials and Methods:**

A cross sectional study was carried out on 227 patients with acute myocardial infarction and demographic data, educational level, marital status, type of transfer to hospital and delay in arrival to hospital were recorded.

**Results:**

35.7% patients arrived during one hour of symptom onset, and 7.9% arrived after 24 hours. Patients having high level education (P = 0.0492) and with a family history of coronary artery disease (P = 0.01) had significantly less delay in arriving to hospital. Age, marital status, gender, and route of transfer to hospital were not related with pre-hospital delay (P > 0.05). Patients thought most common cause of delay in arrival was unawareness of coronary artery disease (38.8%) and self-medication (34.3%).

**Conclusions:**

Increasing awareness of patients about cardiovascular symptoms and their risk factors could be helpful in patient's decision in seeking medical help. So general education via media and primary and middle schools could be helpful.

## 1. Background

Cardiovascular diseases are main cause of mortality and morbidity worldwide (1, 2). Coronary artery diseases (CAD) especially acute myocardial infarction (AMI) need prompt and urgent diagnosis and therapy because one third of deaths caused by AMI occur shortly after symptom onset and before reaching to hospital (2). Medical intervention is necessary in course of treatment of AMI and early use of reperfusion therapy decreases mortality and morbidity of ST-elevation myocardial infarction (STEMI) and fatal arrhythmias, response to thrombolytic is time dependent, initiating treatment during first hour of AMI is crucial for these patients (3-5).

Initiating the reperfusion therapy in less than 60 minutes decreases mortality and morbidity in patients with AMI to a rate of 50% (6), despite this finding, different studies have shown that only a few patients reach hospital during this golden time and it is shown that only 22-44% and sometimes 50% of patients reach hospital during 2 hours after symptom onset (7, 8).

Treatment delay could be because of pre-hospital causes from onset of symptoms till hospitalization of the patient, or in-hospital causes (from hospital admission till initiating the thrombolytic therapy). Pre-hospital causes are main causes of delay in starting reperfusion therapy. Pre-hospital delay is divided in two groups: time from onset of symptoms till decision of the patients to arrive to hospital and time from deciding to seek medical help to arriving to the hospital (2, 3, 9, 10).

## 2. Objectives

This study evaluated the causes of pre-hospital delay just as other studies on effect of different variables such as socioeconomic and personal factors on pre-hospital delay in with patients with AMI.

## 3. Materials and Methods

A cross-sectional analytical study was carried out on patients admitted with AMI during one year in Shahid Mohammadi hospital (General educational hospital, Bandar Abass-Iran). Demographic data, educational level, CAD risk factors, past medical history and family history of CAD, type of arrival to hospital (by EMS, relatives or self) and delay in hospital admission, symptom onset and causes of pre-hospital delay were recorded in a questionnaire. Pre-hospital delay was recorded as less than 1 hour, 1-6, 6-12, 12-18, 18-24, and more than 24 hours. Data were recorded from interview with patients. 240 patients were included in our study and 13 patients were excluded because the duration of pre-hospital delay was not defined in these patients. Data of 227 patients were analyzed using EPI info 2011. Categorical data were expressed as numbers and percentages, continuous data as mean ± standard deviation (SD). The chi-square test was used to analyze the categorical data, and the t tests and analysis of variance were used to examine differences between the different delay groups for continuous variables. Multivariable Logistic regressions were performed to identify the predictors of prehospital delay. Odds ratio (OR) and 95% confidence interval (CI) were calculated and P <0.05 was considered statistically significant.

## 4. Results

**Figure 1.**
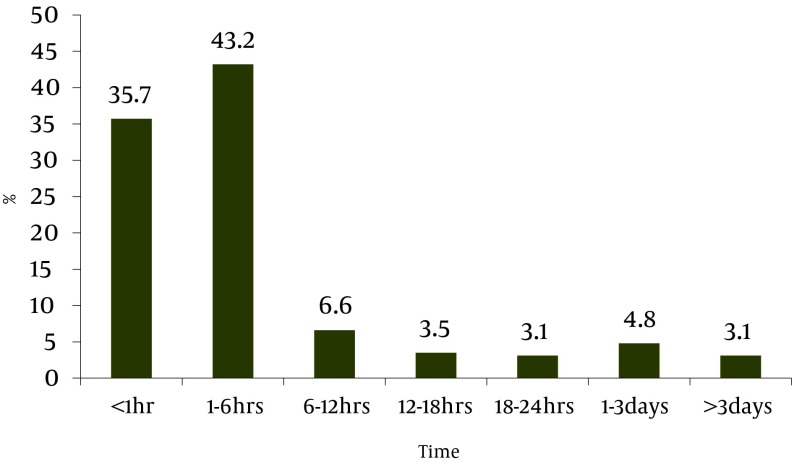
Time Elapsed Between Onset of Symptoms and Hospital Admission

26.9% of 227 patients admitted with diagnosis of AMI were women. Mean age of patients was 57.93 ± 13.08 years and 72.3% were married, 4.4% had university education, while 46% had lower educations and 49.6% were illiterate. Positive history of CAD was present in 22.5% and 2.9% had a positive family history of CAD. Chest pain, sweating and vomiting were seen in 94.3%, 79.7% and 44.5% of patients respectively. Only 35.7% of patients reached to hospital during the first hour on onset of symptoms, and 7.9% reached after 24 hours after symptom onset (Figure 1).

Regarding the importance of initiating therapy during the first hour after symptom onset, patients were divided in two groups: with less than one-hour delay and more than one-hour delay and were compared. Results showed that mean age of patients referring during first hour of symptom onset was less than means age of patients referred with more than one hour of delay (P= 0.07) (Table 1).

**Table 1.** Compare Patients With Delay Less and More Than One Hour

	Delay less than 1 hour (%) N= 81 (35.7%)	Delay more than one hour (%) N= 146 (64.3%)	P Value^a^
**Mean Age**	55.86 ± 12.63	59.08 ± 13.23	0.07
**Age group (yrs)**			
≥ 40 yrs	41.7	58.3	
41-60 yrs	37.7	62.3	0.53
>60 yrs	31.4	68.6	
**Gender**			
Male	35.5	64.5	0.53
Female	36.1	63.9	
**Marital status**			
Married	36.4	63.6	0.42
non married	33.9	66.1	
**Educational level**			
University	50	50	
Diploma and less	42.3	57.7	0.04
Illiterate	27.7	72.3	
**Previous history coronary artery disease**			
Yes	45.1	54.9	0.07
No	33	67	
**Familial history of coronary artery disease**			
Yes	50	50	0.01
No	31.4	68.8	
**Admitted to hospital via**			
EMS	43.4	56.6	0.14
Other	32.6	67.4	

^a^P values of < 0.05 were considered statistically significant

64.5% of men and 63.9% of women had a delay of more than 1 hour (P = 0.53) also 63.6% of married patients and 66.1% of non-married had a delay of more than one hour (P = 0.42). Patients having a higher level of education had a lower rate of pre-hospital delay. Pre-hospital delay more than one hours was more seen in illiterates, low educated and highly educated in 72.3%, 57.7%, and 50% respectively (P = 0.04). Having a positive history of CAD had no correlation with pre-hospital delay (P = 0.07) but in patients with a positive family history of CAD, pre-hospital delay decreased (P = 0.01).

23.5% of patients contacted emergency medical services (EMS) and were brought to hospital by EMS and 56.6% of these patients had a delay of more than one hour and this delay was seen in 67.4% of patients who were brought to hospital via other ways (i.e. by their relative or by themselves) (P = 0.14). In the patients’ opinions, most common causes of pre-hospital delay were their unawareness about their CAD (38.8%), ignoring the symptoms of CAD and self-medications (34.3%), living in farther distance form hospital and lack of suitable transportation (11.4%), lack of equipment and proper first line medications (9.2%) and misdiagnosis of physician (4%). In multivariate regression analysis, there were no independent predictors of prolonged pre-hospital delay (Table 2).

**Table 2.** Predictors of ProlongedPrehospital Delay in Multivariate Logistic Regression Analysis

	Odds ratio	95% confidence interval	P value
**Age**	0.98	0.96-1.00	0.24
**Gender (male/Female)**	0.84	0.44-1.63	0.62
**Educational level(university/ non-university)**	1.76	0.43-7.17	0.42
**Marital status(non-married/ married**	1.04	0.53-2.02	0.90
**Familial history of coronary artery disease(yes/no)**	1.33	0.71-2.50	0.36
**Non-EMS/EMS**	0.57	0.29-1.12	0.10

## 5. Discussion

This study focused on causes of pre-hospital delay in treatment of patients with AMI. A total of 227 patients admitted with AMI were included in this study. Results show that only 35.7% of patients arrived to hospital during the first hour after symptom onset, 43.2% arrived during 1-6 hours and 21.1% arrived after 6 hours after symptom onset, or better to say 64.3% had a pre-hospital delay of more than one hour. Importance of initiating prompt treatment of AMI is proven, so different studies have been carried out on causes of pre-hospital delay. A study on 364131 patients with AMI was carried out for 4 years and pre-hospital delay of more than one hour showed that pre-hospital delay during 1994-1997 was 79.5%, 79.8%, 79.3% and 79.9% respectively (11). Similar studies show similar results. Sari had a delay of more than hour in 69% of patients with AMI.2 Song has reported a delay of more than 2 hours in 55.4% and more than 6 hours in 20.3% patients (12). In a similar study in Pakistan 33.9% of patients had a delay of more than 6 hours and only 36% of patients arrived before two hours of symptom onset.6 and McGinn showed in his study on 18928 patients that 49.5% had a delay of more than 4 hours from symptom onset to arriving to the hospital (13). Two other studies showed median of pre-hospital delay in four hospitals in London was 2 hours and in five hospitals in USA this time was 4.25 hours (3, 5).

High level of education and a positive family history of CAD were significantly correlated with a short delay in arrival to hospital after symptoms onset. Unawareness of CAD risk factors and ignoring the symptoms were seen in 73.1% in our study, which shows that patients have low level of information about CAD and this issue is correlated with low education of patients and negative family history of CAD. Previous studies have shown that low level of education and unawareness about symptoms of CAD causes a delay in arrival to hospital (2, 7, 12).

Other studies have not found a definite correlation between low level of education and delay in arrival to hospital but have shown that awareness of patients about CAD symptoms has decreased the time of arrival to hospital (3, 5, 6). A history of CAD in patient or family members could increase the awareness of patients about symptoms of CAD, noticing them. Our study showed that having a family history of CAD decreased the delay time in hospital arrival significantly (P = 0.01). Our study showed that first heart attack or recurrent heart attack is not correlated with a decrease in arrival to hospital, just as other similar studies (5, 7). but it has been shown that patients who arrive earlier to hospital had become aware of CAD either by their physician or their family members (7). Also Sogo showed that previous experience of the patient had a better effect in diagnosing the disease, but family history was not effective enough (12), on contrary Sari showed that a previous experience of CAD shortens the arrival time (2).

Age, gender and marital status are some factors which are thought to be effective on time of arrival to hospital. Our study has not shown a significant correlation between these variables and arrival to hospital, but other studies show advanced age is directly correlated with increased arrival time, (2, 3, 7, 8, 13-15) but our study and studies similar have shown age and time of arrival are not correlated (5, 6). Banks in USA and Khan in Pakistan believe that gender is not correlated with decreased arrival time to hospital (5, 6), but other studies show that women have increased pre-hospital delays (2, 7, 8, 13, 15). Marital status could be directly correlated with lifestyle and quality of life. Sari and Khan believe that pre-hospital delay is not correlated with marital status like our study (2, 6), but Perkins-Porras, Banks and Saczynski show that single patients have greater delay time (3, 5, 15). On time transport to hospital decreases delay time. It seems that type of transport is associated with decreased delay time, and EMS decreases pre-hospital time (3, 5, 10, 13, 14), but our study doesn’t show any correlation with EMS services and decrease in pre-hospital delay. Lack of decision by patient for using EMS or delay in ambulance arrival because of incorrect addresses or few numbers of available ambulances could be correlated with this issue.

According to our study’s results and regarding previous studies it seems the main controllable factor in reducing pre-hospital delay in AMI treatment is increasing awareness of patients from cardiovascular alarming symptoms so that patients can decide promptly when to see a doctor. General education through media (radio, television, newspaper) and primary and middle schools is proposed. On the other hand, special attention should be paid to EMS services so that patients can be transferred to hospital with least possible delay. According to our study a large number of patients come to hospital during 1-6 hrs after onset of symptoms, it seems that educating the patients and improving EMS services can decrease this time.
